# Posttraumatic Stress Disorder and Community Collective Efficacy following the 2004 Florida Hurricanes

**DOI:** 10.1371/journal.pone.0088467

**Published:** 2014-02-11

**Authors:** Robert J. Ursano, Jodi B. A. McKibben, Dori B. Reissman, Xian Liu, Leming Wang, Robert J. Sampson, Carol S. Fullerton

**Affiliations:** 1 Department of Psychiatry, Uniformed Services University of the Health Sciences, Bethesda, Maryland, United States of America; 2 National Institute for Occupational Safety and Health, Office of the Director, Washington, D.C., United States of America; 3 Department of Sociology, Harvard University, Cambridge, Massachusetts, United States of America; Harvard Medical School, United States of America

## Abstract

There is a paucity of research investigating the relationship of community-level characteristics such as collective efficacy and posttraumatic stress following disasters. We examine the association of collective efficacy with probable posttraumatic stress disorder and posttraumatic stress disorder symptom severity in Florida public health workers (n = 2249) exposed to the 2004 hurricane season using a multilevel approach. Anonymous questionnaires were distributed electronically to all Florida Department of Health personnel nine months after the 2004 hurricane season. The collected data were used to assess posttraumatic stress disorder and collective efficacy measured at both the individual and zip code levels. The majority of participants were female (80.42%), and ages ranged from 20 to 78 years (median = 49 years); 73.91% were European American, 13.25% were African American, and 8.65% were Hispanic. Using multi-level analysis, our data indicate that higher community-level and individual-level collective efficacy were associated with a lower likelihood of having posttraumatic stress disorder (OR = 0.93, CI = 0.88–0.98; and OR = 0.94, CI = 0.92–0.97, respectively), even after adjusting for individual sociodemographic variables, community socioeconomic characteristic variables, individual injury/damage, and community storm damage. Higher levels of community-level collective efficacy and individual-level collective efficacy were also associated with significantly lower posttraumatic stress disorder symptom severity (b = −0.22, p<0.01; and b = −0.17, p<0.01, respectively), after adjusting for the same covariates. Lower rates of posttraumatic stress disorder are associated with communities with higher collective efficacy. Programs enhancing community collective efficacy may be an important part of prevention practices and possibly lead to a reduction in the rate of posttraumatic stress disorder post-disaster.

## Introduction

State and local public health workers play a critical role as first responders. Concern over public health response to natural disasters increased in the aftermath of the 2004 Asian tsunami, Hurricane Katrina, and the 2010 earthquakes in Haiti and Chile. Public health workers living in disaster-affected communities experience the direct effect of disasters, and at the same time are responsible for providing care to others. Public health workers exposed to disasters have high rates of acute and longer-term posttraumatic distress and posttraumatic stress disorder (PTSD) [1]–[4]. Few studies have addressed the psychological consequences of disaster in a large population of public health workers [2], [5]–[8]. Further, the possibility of community-level characteristics such as collective efficacy, defined as social cohesion among neighbors along with their willingness to intervene for the common good [9], mitigating the impact of such psychological consequences following disasters has not been addressed.

Multiple community characteristics influence health outcomes [9], [10]. The majority of studies of disaster mental health, which address neighborhood and social processes, measure and analyze them as individual-level variables [11], [12]. Collective efficacy can be both an individual-level perception and a community-level capacity. At the community level, the willingness of community members to intervene for the common good depends on mutual trust and solidarity among neighbors [13]. Collective efficacy is associated with neighborhood poverty, violence, and disadvantage [9], [14]–[18]. Specifically, increases in community collective efficacy are related to lower levels of depressive symptoms [19] and decreases in neighborhood crime [9], [16], [20]. Mental health outcomes have also been shown to be positively influenced by the presence of collective efficacy. In particular, intimate partner violence and antisocial behavior in adolescence have been shown to be less prevalent in communities with higher levels of collective efficacy [16], [17].

While a number of disaster mental health studies have measured various aspects of collective efficacy at the individual level, to our knowledge, none have examined it at the community level. Further, collective efficacy has only been assessed at the individual level in post-disaster settings. Perceptions of collective efficacy were examined one year after the small community of Buffalo Creek, Colorado was destroyed by a forest fire and then a flood within a 2-month period in 1996 [11]. Perceived social support, resource depletion and psychological distress 3–8 weeks post-disaster predicted perceived collective efficacy at one year. Results suggest that social resources, i.e., social support and perceptions of collective efficacy, had buffering effects against psychological distress under conditions of high resource loss following a disaster [11].

The 2004 Florida hurricane season was unprecedented. Four hurricanes (Charley, Frances, Ivan, and Jeanne) and one tropical storm (Bonnie) made landfall within a period of seven weeks [21], [22]. The $4.85 billion in costs incurred for hurricane relief accounted for nearly 88% of the total disaster aid in 2004 [23]. The 2004 hurricane season provided a unique opportunity to examine public health workers of the Florida Department of Health (FDOH) who experienced both personal hurricane-related injuries and high levels of community storm damage within communities. This study examines the relationship of both community-level and individual-level collective efficacy to posttraumatic stress symptoms and the prevalence of PTSD in this population of FDOH public health workers nine months post-hurricanes. To our knowledge, this is the only disaster mental health study to examine collective efficacy at the community level. It is also the only study to use individual collective efficacy (perceived collective efficacy) to predict PTSD. Understanding the relationship between community-level factors and mental health has important implications for the allocation of resources across communities.

## Methods

### Ethics Statement

The study was conducted in accordance with the ethical standards and approval of the Institutional Review Board, Uniformed Services University of the Health Sciences, Bethesda, MD. Participation was voluntary. Questionnaires and a project description were distributed to FDOH employees using the personnel e-mail distribution lists. All participants indicated agreement to participate by completing a questionnaire that was transmitted electronically and anonymously.

### Participants and Procedures

In June 2005, approximately 9 months after the 2004 hurricane season, FDOH employees were asked to report their work and personal experiences during and since the 2004 hurricane season. Two versions of the questionnaire (i.e., A and B) were distributed randomly so that each potential participant received either version. Questionnaire versions contained some of the same items and some unique items, with version A focusing on mental health items.

Of an estimated 8564 FDOH personnel who worked during the 2004 hurricanes and were available at the time of the survey, we were able to contact and invite 6637 individuals to participate. After reading a description of the study and the informed consent, 4323 agreed to participate, and completed and returned the questionnaire (Version A = 2249; Version B = 2074), with an estimated response rate of 65.1%. This study used respondents completing Version A. Ages of the participants ranged from 20 to 78 years (median = 49 years). The majority were female (80.42%, n = 1787) and currently married (66.52%, n = 1482). The majority were White (73.91%, n = 1623), 13.25% (n = 291) were African American, 8.65% (n = 190) were Hispanic, and 4.19% (n = 92) other. Nearly half of the participants had less than a BA/BS degree (48.88%, n = 1091). Prior trauma exposure only as a child was reported by 5.7% (n = 128) of participants, 20.8% (n = 464) reported prior trauma exposure only as an adult, and 14.8% (n = 330) reported prior trauma exposure both as an adult and as a child.

### Measures

#### Posttraumatic Stress Disorder (PTSD)

PTSD symptom severity scores and probable PTSD were assessed with the 17-item PTSD Checklist (PCL-17) [24]. The PCL-17 lists all symptoms of PTSD outlined in the DSM-IV. Respondents rated how much they had been bothered by each problem in the past month on a scale ranging from 1 (not at all) to 5 (extremely). Each question was worded so as to be related to the respondent's experience with the hurricanes. Responses were summed to produce PTSD symptom severity scores ranging from 17 to 85.

Studies in primary care settings with populations similar to ours have validated a PCL-17 score of 30 or greater as indicative of probable PTSD (sensitivity = .78–.82, specificity = .71–.76), positive and negative likelihood ratios of 3.40 and 0.24, respectively, and Cronbach's alpha was .96 for the total PCL score [25], [26]. In this study, participants were rated as having probable PTSD if they had scores of 30 or greater and also met the following DSM-IV symptom distribution criteria: one intrusion, three avoidance, and two hyperarousal symptoms, each present at the level of moderate or higher during the previous month.

#### Collective efficacy

Collective efficacy was assessed with the 10-item scale (range 10–50) employed by Sampson and colleagues [9]. The scale has five items in each of two domains: informal social control and social cohesion/trust. Each individual's response to the two five-item, five-point Likert scales (ranging from very likely to very unlikely and strongly disagree to strongly agree) were summed to a total score for individual level collective efficacy. Informal social control includes five items that ask how likely it would be that their neighbors could be counted on to intervene if: a) children were skipping school and hanging out on a street corner; b) children were spray painting graffiti on a local building; c) children were showing disrespect to an adult; d) a fire broke out in front of their house; and e) if a fire station closest to their home was threatened with budget cuts. The social cohesion/trust scale includes five items that assess the extent to which participants agreed that in their home neighborhood: a) people are willing to help their neighbors; b) it is a close-knit neighborhood; c) people can be trusted; d) people generally get along with each other; and e) people share the same values. Higher scores indicate greater collective efficacy. Sampson and colleagues [9] demonstrated high between-neighborhood reliability (ranging from 0.80 to 0.91) across 343 neighborhoods in Chicago, IL. There was a strong association between social cohesion and informal social control across neighborhoods (r = 0.80, p<0.001), suggesting these scales were measuring aspects of the same latent construct.

Community level collective efficacy was assessed using zip codes to define the community units. For each zip code, the sample mean of those individuals in the zip code was obtained and rescaled as a centered variable about the grand mean of the entire sample. Since a zip code represents a collection of people and institutions that occupy a unique subsection of a geographic location, each zip code is sufficiently externally heterogeneous and internally homogeneous to be used in multilevel analyses. Given this design, 825 zip codes served as the level-two unit in this study.

#### Individual hurricane injury/damage

Injury/damage at the time of the hurricanes was assessed as an individual-level variable by asking participants whether they had experienced any of the following six events during each of the five hurricanes: loss of electrical power; damage to vehicle; injury or harm to self; injury or harm to spouse/significant other; and injury/harm to children or injury/harm to pets. Those reporting at least two of the events during the five hurricanes were considered to have high hurricane-related injury/damage (n = 1093, 58.14%).

#### Community hurricane damage

Using FEMA county data for all five storms [23], we identified the zip code level of FEMA public and individual assistance received. Each zip code was scored based on its highest community storm damage across the five storms to index the level of individual and public assistance received. We combined levels to create five levels of public assistance and, therefore, community storm damage. The level of community storm damage ranged from none (0) to individual assistance only (1) to increasing levels of public assistance with FEMA categories A to G (scored 2, 3 and 4). This level-two variable was then centered.

#### Socioeconomic characteristics

Ten zip code specific census measures assessed socioeconomic characteristics (Table 1). Following Sampson's model [9], three community-level factor scores, concentrated disadvantage, immigrant concentration, and residential stability, were extracted from the ten zip code specific census measures. We used a principal factor analysis with squared multiple correlations (SMC) for the prior communality estimates. Both orthogonal and oblique rotations were applied. The oblique rotated factor pattern was highly consistent with those reported by Sampson and associates [9] (Table 1). Factor 1, concentrated disadvantage, had an eigenvalue of 3.94, with high loadings for poverty, receipt of public assistance, unemployment, female-headed families, density of children, percentage of Black residents, and the percentage of owner-occupied homes. Factor 2, immigrant concentration, captured two variables with high loadings, the percentage of Latinos and the percentage of foreign-born individuals. Factor 3, residential stability, had one variable with a high loading, the percentage of persons living in the same house for the past five years. The three factors were constructed as standardized scores with a mean of 0 and a standard deviation of 1. These factors were used as level-two control variables in the multilevel analyses.

**Table 1:** Oblique rotated factor pattern loadings (≥0.60) in 825 Florida zip codes.

Variable		Factor loading
Concentrated disadvantage	Below poverty line	0.86
	On public assistance	0.72
	Female-headed families	0.85
	Unemployed	0.73
	Black	0.90
	Owner-occupied house	0.64
Immigrant concentration	Latino	0.95
	Foreign-born	0.90
Residential stability	Same house as five years ago	0.64

### Statistical Analysis

Potential individual- and community-level risk factors for higher PTSD symptom severity scores and probable PTSD at 9 months post-hurricane in FDOH employees were analyzed using a multilevel modeling approach. The level 1 unit was individuals (n = 1800) and the level 2 unit was zip code-defined communities (n = 825). All analyses excluded missing cases across all covariates (n = 1880). Statistical analyses were conducted using SAS software Version 9.2 [27]. Specifically, SAS PROC MIXED and SAS PROC GLIMMIX were used. Both apply empirical Bayesian approaches for handling low reliability in some of the level-2 units [28], [29].

#### PTSD symptom severity

Random coefficient analyses were used to evaluate the associations with PTSD symptoms. The individual-level collective efficacy predictor was considered in the presence of both individual (sex, race, age, education, marital status, and individual injury/damage) and community-level (concentrated disadvantage, immigrant concentration, residential stability, and community storm damage) covariates. The interaction between injury/damage and individual-level collective efficacy, and the interaction between injury/damage and community storm damage were included as additional fixed effects. We considered three random effects for the intercept, for the slopes of injury/damage, and for the participants within communities. The degree of clustering within zip codes was assessed by the intra-communities correlation [30]. We applied the same multilevel approach for community-level collective efficacy, with individual collective efficacy replaced by community-level collective efficacy. We constructed a multilevel model by including all of the aforementioned covariates.

#### Probable PTSD

Random intercept analyses were used to evaluate the associations with probable PTSD. The same fixed effects discussed above were included in these analyses as well. However, only one random effect for the intercept was included. As above, analyses for both individual-level and community-level collective efficacy were conducted including all of the aforementioned covariates.

The intra-communities correlation was calculated for these models as well. The median odds ratio (MOR) was calculated to translate the community-level variance to an odds ratio scale that would be directly comparable to the odds ratios of individual-level or community-level fixed effects [31]. The MOR is defined as the median value of the odds ratio between the community at the lowest risk and the community at the highest risk.

Since odds ratios only provide indirect information on covariates' effects and the use of multiple interactions and centered covariates further complicate interpretation [32], predicted probabilities of probable PTSD were calculated for five collective efficacy scores (10, 20, 30, 40 and 50) by two levels of injury/damage (low and high), using the results of the final multilevel logistic regression models. The parameter estimates were marginalized to produce estimates of the probabilities [31]. In deriving the marginalized probabilities, values of all other independent variables were fixed as sample means, so that probabilities of probable PTSD across the aforementioned collective efficacy and injury/damage levels could be efficiently compared.

## Results

Nine months after the 2004 hurricanes, high levels of individual injury/damage and high levels of community storm damage were reported in this group of FDOH workers. Specifically, 58.14% (n = 1093) experienced high levels of personal injury/damage, and the average level of community storm damage was 1.51 (SD = 1.14) (Table 2). On a scale ranging from 17 to 85, the average total PTSD symptom severity score was 23.78 (SD = 9.13). Approximately four percent (4.36%, N = 82) of FDOH employees met PTSD criteria using the PCL diagnostic algorithm. The average scores for individual-level and community-level collective efficacy were 36.07 (SD = 7.65) and 36.12 (SD = 4.29), respectively. After accounting for missing data across the predictor variables, 1880 cases remained for all analyses below.

**Table 2:** Sample characteristics for collective efficacy, PTSD, individual and community factors (n = 1880).

Sample characteristics		Mean or % (SD)
Collective efficacy	Individual-level collective efficacy, mean (SD)	36.07 (7.65)
	Community-level collective efficacy, mean (SD)	36.12 (4.29)
PTSD	PTSD symptom severity score, mean (SD)	23.78 (9.13)
	Probable PTSD (present), % (SD)	4.36 (0.20)
Demographics/individual factors	Sex (female), % (SD)	81.91 (0.39)
	Age, mean, (SD)	47.53 (10.30)
	Race/ethnicity (white), % (SD)	73.40 (0.44)
	Education (<BA/BS degree), % (SD)	50.37 (0.50)
	Marital status (married), % (SD)	65.48 (0.48)
	Injury/damage (high), % (SD)	58.14 (0.49)
Community factors	Community storm damage, mean (SD)	1.51 (1.14)
	Concentrated disadvantage, mean (SD)	0.01 (0.93)
	Immigrant concentration, mean (SD)	−0.06 (0.89)
	Residential stability, mean (SD)	0.01 (0.89)

### PTSD symptom severity

Two random coefficient effects analyses were conducted to evaluate the associations between a) individual-level collective efficacy and PTSD symptom severity and b) community-level collective efficacy and PTSD symptom severity. These relationships were considered while adjusting for the aforementioned individual sociodemographic variables, community socioeconomic characteristic variables, individual injury/damage, community storm damage, the interaction between injury/damage and collective efficacy, and the interaction between injury/damage and community storm damage.

#### Individual-level collective efficacy

Beginning with a model containing all covariates, analyses revealed that an increase in individual-level collective efficacy was associated with a significant decrease in PTSD symptom severity. Also, having high injury/damage was related to a significant increase in PTSD symptom severity. We also examined the model after removing the nonsignificant two interactions and three socioeconomic characteristics. Making this change to the model did not significantly change the model chi-square and the parameter estimates remained essentially unchanged. We used the full model because of the theoretical relevance of these variables to PTSD symptom severity and the preference for using the same approach as that employed by Sampson and colleagues [9]. In the selected full model, after adjusting for all covariates, a one point increase in individual-level collective efficacy was associated with a 0.17 point decrease (p<0.01) in PTSD symptom severity (Table 3). The intra-communities correlation for the individual-level efficacy model was 0.067. Model χ^2^ = 231.80 (p<0.01).

**Table 3:** Parameter estimates of two multilevel linear regression models on PTSD symptom severity (n = 1880).

Variable		Individual-level collective efficacy models	Community-level collective efficacy models
		Full model	Full model
Fixed effect	Intercept	23.75***	23.82***
	Collective efficacy (ind/coll)	−0.17***	−0.22***
	Sex	0.49	0.30
	Age	−0.01	−0.02
	Race/ethnicity	0.24	0.41
	Education	−0.80*	−0.80*
	Marital status	−1.18**	−1.47**
	Injury/damage	2.39***	2.57***
	Community storm damage	0.37*	0.43*
	Concentrated disadvantage	0.27	0.29
	Immigrant concentration	−0.25	−0.37
	Residential stability	0.09	0.06
	Collective efficacy x injury	−0.06	−0.17
	Storm x injury	0.36	0.17
Random effect	Between communities (τ00)	5.12***	5.32***
	Slope of coleff^a^/injury^b^ (τ11)	0.07***	4.25
	Between intercept & slope (τ10)	−0.57***	8.67***
	Within communities (σ^2^)	71.46***	75.31***
	ICC	0.07	0.07
Model χ^2^		231.80***	198.70***

a Individual-level collective efficacy model.

b Community-level collective efficacy model.

* p<0.10;

** p<0.05;

*** p<0.01.

#### Community-level collective efficacy

In a model with all covariates included, analyses revealed that an increase in community-level collective efficacy was associated with a significant decrease in PTSD symptom severity. Further, having high injury/damage was associated with an increase in PTSD symptom severity. We examined the model after removing the nonsignificant two interactions and three socioeconomic characteristics. This modification to the model did not significantly change the model chi-square and the parameter estimates remained essentially unchanged. We selected the full model because of the theoretical relevance of these variables to PTSD symptom severity and the opportunity to replicate Sampson's (9) approach. In the full model, including all covariates, a one point increase in community-level collective efficacy was associated with a 0.22 point decrease (p<0.01) in PTSD symptom severity (Table 3). The intra-communities correlation for the community-level efficacy model was 0.066 (Model χ^2^ = 198.70, p<0.01).

### Posttraumatic Stress Disorder

Using random-intercept models with the same covariates, we examined the relationship of individual- and community-level collective efficacy to a probable PTSD diagnosis.

#### Individual-level collective efficacy

Using a model that included all covariates, analyses revealed that an increase in individual-level collective efficacy was associated with a decreased probability of having probable PTSD (OR = 0.94, CI = 0.92–0.97). Further, having high injury/damage increased the probability of being diagnosed with probable PTSD (OR = 2.63, CI = 1.33–5.21).

Eliminating the two interactions and three community socioeconomic characteristics produced a significant change in the model chi-square, although the parameter estimates remained essentially unchanged. Given the stronger predictive power, and in line with our previous approach, we selected the full model. In the final model containing all covariates, for every point increase in individual-level collective efficacy, the odds of having probable PTSD decreased by 6% (OR = 0.94, CI = 0.92–0.97) (Table 4). The intra-communities correlation for individual-level collective efficacy model was 0.14 (Model χ^2^ = 354.94).

**Table 4:** Parameter estimates of multilevel logistic regression models for individual-level collective efficacy on probable PTSD (n = 1880).

Individual-level collective efficacy models					
Variable		Full model b(SE)	Full model OR (95% CI)	Reduced model b(SE)	Reduced model OR (95% CI)
Fixed effect	Intercept	−3.37 (0.15)	–	−3.29 (0.14)	–
	Collective efficacy	−0.06 (0.01)	0.94 (0.92–0.97)	−0.05 (0.01)	0.95 (0.93–0.98)
	Sex	−0.19 (0.38)	0.83 (0.40–1.74)	−0.21 (0.37)	0.81 (0.39–1.68)
	Age	−0.02 (0.01)	0.98 (0.96–1.01)	−0.02 (0.01)	0.98 (0.96–1.01)
	Race/ethnicity	0.34 (0.34)	1.41 (0.73–2.74)	0.33 (0.34)	1.39 (0.72–2.68)
	Education	−0.44 (0.28)	0.65 (0.37–1.12)	−0.45 (0.28)	0.64 (0.37–1.10)
	Marital status	−0.27 (0.27)	0.77 (0.45–1.29)	−0.27 (0.26)	0.77 (0.46–1.29)
	Injury/damage	0.97 (0.35)	2.63 (1.33–5.21)	0.82 (0.32)	2.27 (1.21–4.27)
	Community storm damage	0.14 (0.12)	1.15 (0.91–1.45)	0.14 (0.11)	1.15 (0.92–1.43)
	Concentrated disadvantage	−0.14 (0.15)	0.87 (0.64–1.17)		
	Immigrant concentration	0.09 (0.14)	1.09 (0.83–1.43)		
	Residential stability	0.40 (0.18)	1.50 (1.05–2.13)		
	Collective efficacy x injury	0.06 (0.04)^a^	–		
	Storm x injury	−0.08 (0.29)^b^	–		
Random effect	Between communities (τ00)	0.51 (0.28)		0.52 (0.27)	
	Median odds ratio (95% CI)		2.01 (1.77–2.34)		2.03 (1.77–2.36)
	ICC	0.14		0.14	
Model χ^2^		354.94		212.52	
Difference in model χ^2^				142.42**	

p<0.05;

** p<0.01.

a Wald χ^2^ = 1.50, df = 1, ns.

b Wald χ^2^ = −0.28, df = 1, ns.

**Figure 1:**
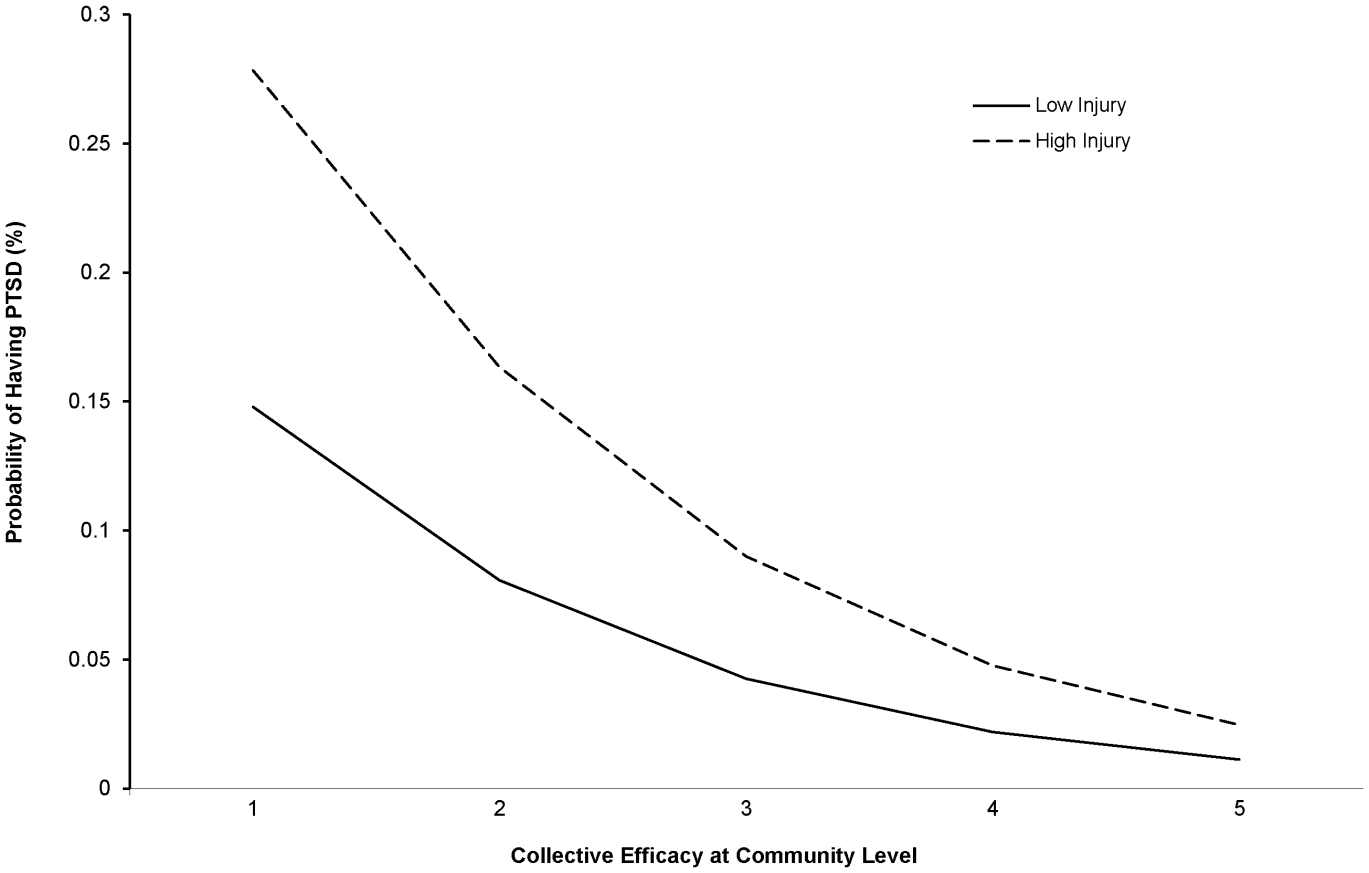
Changes in probability of having PTSD over two injury/damage groups and five community-level efficacy levels.

Another way to highlight the effects of collective efficacy on PTSD is to compare the predicted probabilities of having PTSD for different subgroups of injury/damage, while controlling for all other covariates. Table 5 displays the estimated marginalized probabilities of having PTSD across the five levels of individual-level collective efficacy by the two injury/damage subgroups (low and high). Figure 1 plots these changes in probabilities for the community-level. However, the graph is quite similar for the individual-level. Persons with higher individual-level collective efficacy have a considerably lower chance of having PTSD than do their counterparts with lower levels of collective efficacy, irrespective of their level of injury/damage.

**Table 5:** Estimated marginalized probabilities of probable PTSD across five collective efficacy scores by level of injury/damage.

	Individual-level		Community-level	
Level of collective efficacy	Low injury/damage	High injury/damage	Low injury/damage	High injury/damage
10	0.093	0.182	0.148	0.278
20	0.060	0.120	0.081	0.163
30	0.038	0.078	0.043	0.090
40	0.024	0.050	0.022	0.048
50	0.015	0.031	0.011	0.025

Of those with high injury/damage, the probability of having PTSD is expected to be 0.12 if a person has a lower level of individual-level collective efficacy (score = 20). This risk decreases sharply to 0.05 if he or she has a higher level individual-level collective efficacy (score = 40), which is a 59% reduction. For those with lower injury/damage, the risk of having PTSD is expected to drop from 0.06 with a lower level of individual-level collective efficacy to 0.02 with a higher level of individual-level collective efficacy, which is a 67% reduction.

#### Community-level collective efficacy

In a model that contains all covariates, analyses revealed that an increase in community-level collective efficacy was associated with a decreased probability of having probable PTSD (OR = 0.93, CI = 0.88–0.98). Further, having high injury/damage increased the probability of being diagnosed with probable PTSD (OR = 2.29, CI = 1.19–4.39).

In contrast to the analyses described above, eliminating the two interactions and three community socioeconomic characteristics produced a significant change in the model chi-square. However, the parameter estimates remained essentially unchanged. As such, given the strong statistical power and potential theoretical relevance of the removed variables, we elected to emulate Sampson's approach [9] and retain all covariates in the final models. The intra-communities correlation for the community-level collective efficacy model was 0.13 (Model χ^2^ = 245.73). In the final model, every one point increase in community-level collective efficacy reduced the odds of having PTSD by 7% (OR = 0.93, CI = 0.88–0.98) (Table 6).

**Table 6:** Parameter estimates of multilevel logistic regression models for community-level collective efficacy on probable PTSD (n = 1880).

Community-level collective efficacy models					
Variable		Full model b(SE)	Full model OR (95% CI)	Reduced model b(SE)	Reduced model OR (95% CI)
Fixed effect	Intercept	−3.32 (0.14)	–	−3.26 (0.14)	–
	Collective efficacy	−0.07 (0.03)	0.93 (0.88–0.98)	−0.07 (0.03)	0.93 (0.89–0.98)
	Sex	−0.24 (0.38)	0.79 (0.38–1.64)	−0.25 (0.37)	0.78 (0.38–1.62)
	Age	−0.02 (0.01)	0.98 (0.96–1.01)	−0.02 (0.01)	0.98 (0.96–1.01)
	Race/ethnicity	0.40 (0.34)	1.50 (0.77–2.91)	0.38 (0.34)	1.46 (0.76–2.82)
	Education	−0.43 (0.28)	0.65 (0.38–1.13)	−0.43 (0.28)	0.65 (0.38–1.12)
	Marital status	−0.36 (0.27)	0.70 (0.42–1.18)	−0.33 (0.26)	0.72 (0.43–1.21)
	Injury/damage	0.83 (0.33)	2.29 (1.19–4.39)	0.85 (0.32)	2.34 (1.25–4.39)
	Community storm damage	0.14 (0.12)	1.16 (0.92–1.46)	0.14 (0.11)	1.15 (0.92–1.43)
	Concentrated disadvantage	−0.14 (0.15)	0.87 (0.65–1.17)		
	Immigrant concentration	0.08 (0.14)	1.08 (0.83–1.41)		
	Residential stability	0.41 (0.18)	1.51 (1.06–2.15)		
	Collective efficacy x injury	−0.12 (0.18)	–		
	Storm x injury	−0.02 (0.08)	–		
Random effect	Between communities (τ00)	0.51 (0.27)		0.52 (0.26)	
	Median odds ratio (95% CI)		1.95 (1.72–2.25)		1.97 (1.73–2.28)
	ICC	0.13		0.14	
Model χ^2^		245.73		159.76	
Difference in model χ^2^				85.97**	

p<0.05;

** p<0.01.

Table 5 displays the estimated marginalized probabilities of having PTSD across five levels of community-level collective efficacy by the two injury/damage levels, and Figure 1 plots these changes in the probabilities. This figure illustrates that those residing in a community with higher community-level collective efficacy, regardless of the level of injury, have a considerably lower chance of having PTSD than do their counterparts residing in a community with a lower level of community-level collective efficacy.

Of those with high injury/damage, the probability of having probable PTSD is expected to be 0.16 if a person resides in a community with a low level of community-level collective efficacy (score = 20). This risk decreases sharply to 0.05 if he or she resides in a community with a higher level of community-level collective efficacy (score = 40), which is a 69% reduction. Similarly, for those with lower injury/damage, the risk of having PTSD is expected to drop from 0.08 with a lower level of community-level collective efficacy to 0.02 with a higher level of community-level collective efficacy, which is a 75% reduction.

## Discussion

The health of first responders, including public health workers, is critical to sustaining a community's health. Recent experiences with September 11, Hurricane Katrina, and concerns of an Asian influenza pandemic further emphasize this issue. The professional role of disaster workers can be both a risk and a resilience factor. Disaster workers have training to protect themselves and reduce stress, but also can experience both direct and secondary vicarious traumatic stress [33], [34]. Disaster workers as a group show a pattern of both acute and long term distress and dysfunction [2], [3], [35]–[38]. In particular, public health workers experience acute and longer-term PTSD [1]–[3], [38]. In addition, they may, as in this study, live in the affected community. Their communities can be an additional resource promoting resilience or an additional stressor. The FDOH disaster workers reported high levels of individual injury/damage (58.14%, n = 1093) and high community storm damage (M = 1.51, SD = 1.14). Approximately 4% of FDOH employees met criteria for hurricane-related PTSD. This rate is similar to the conditional probability of PTSD (3.8%) found in populations exposed to natural disasters [39].

Community resources are important for disaster mental health outcomes [11], [38]. However, most studies assess community resources or characteristics at the individual level. In contrast, in the present study, we examined collective efficacy at both the individual level (the perception of collective efficacy) and at the community level using zip codes to define the community units. Our data indicate that disaster workers who lived in neighborhoods with higher community-level collective efficacy had a lower likelihood of probable PTSD, even after adjusting for individual sociodemographic variables, community socioeconomic characteristic variables, individual injury/damage, and community storm damage. This was also true when we examined collective efficacy as an individual's perception of their community. Those public health workers who reported higher individual-level collective efficacy, which we consider perceived collective efficacy, had a lower risk of PTSD. Higher levels of community-level collective efficacy and individual-level collective efficacy were also associated with significantly lower PTSD symptom severity after adjusting for the same covariates. The finding of lower PTSD associated with higher collective efficacy suggests that communities with higher collective efficacy may have characteristics which foster recovery and lower stress associated with disasters. Communities with higher collective efficacy may promote experiences of safety, calming, optimism, and social support [40]. In such communities, members are more likely to have lower exposure to chronic adversities, work together to make resources available for rebuilding, and provide mutual support and assistance. In addition, there may be greater use of health care that can prevent or mitigate disorders such as PTSD. Each of these may enhance recovery from acute stress and lead to lower rates of PTSD.

The costs of posttraumatic stress and PTSD are significant [41]–[44]. Following Hurricanes Katrina and Rita, the cost of adequate mental health response for the storm-affected population of 11 million people was $1,133 per person, or $12.5 billion in total [45]. Our study indicates that after a severe storm, comparing a high collective efficacy community (score = 40; anticipated rate of PTSD is 4.8%) to a low collective efficacy community (score = 20; anticipated rate of PTSD is 16.3%), there is a difference of 11.5% in expected rates of PTSD. If this difference in rates of PTSD were similar for the entire disaster-exposed population, the savings in costs through enhanced collective efficacy could be substantial; however, such a comparison must take into account potential differences in rates of PTSD in public health workers and the general population.

The present findings must be interpreted in terms of several methodological considerations. Since this is a cross-sectional study, further research using longitudinal designs is recommended. Since this is a study of public health workers, generalization of findings to other populations is limited and requires further study. Since the sample was subdivided into zip codes, the sample size may affect the representativeness of the zip codes. Zip codes are being used as a proxy for neighborhood. While this is for the most part a reasonable choice, it is plausible that in some cases zip codes will cross neighborhoods.

To our knowledge, this is the first study to demonstrate the significant relationship of community-level collective efficacy to posttraumatic stress disorder. Although these findings are cross-sectional, they suggest that programs that enhance neighborhood cohesion by introducing new funds, building new residences, and altering behaviors could have significant implications for prevention practices and possibly lower rates of PTSD post-disaster. Moreover, intervening at the community level is often cost-effective and practical, and may reach individuals who may not seek or have available individual interventions post-disaster.
